# Chlorotoxin binds to both matrix metalloproteinase 2 and neuropilin 1

**DOI:** 10.1016/j.jbc.2023.104998

**Published:** 2023-06-30

**Authors:** Sándor Farkas, Daniel Cioca, József Murányi, Péter Hornyák, Attila Brunyánszki, Patrik Szekér, Eszter Boros, Patrik Horváth, Zoltán Hujber, Gábor Z. Rácz, Noémi Nagy, Rebeka Tóth, László Nyitray, Zalán Péterfi

**Affiliations:** 1 VRG Therapeutics Ltd, Budapest, Hungary; 2 Department of Biochemistry, ELTE Eötvös Loránd University, Budapest, Hungary

**Keywords:** chlorotoxin, matrix metalloproteinase, neuropilin, annexin, protein–protein interaction, vascular endothelial growth factor, enzyme inhibitor, flow cytometry, protein targeting, glioblastoma

## Abstract

Chlorotoxin (CTX), a scorpion venom–derived 36-residue miniprotein, binds to and is taken up selectively by glioblastoma cells. Previous studies provided controversial results concerning target protein(s) of CTX. These included CLC3 chloride channel, matrix metalloproteinase 2 (MMP-2), regulators of MMP-2, annexin A2, and neuropilin 1 (NRP1). The present study aimed at clarifying which of the proposed binding partners can really interact with CTX using biochemical methods and recombinant proteins. For this purpose, we established two new binding assays based on anchoring the tested proteins to microbeads and quantifying the binding of CTX by flow cytometry. Screening of His-tagged proteins anchored to cobalt-coated beads indicated strong interaction of CTX with MMP-2 and NRP1, whereas binding to annexin A2 was not confirmed. Similar results were obtained with fluorophore-labeled CTX and CTX-displaying phages. Affinity of CTX to MMP-2 and NRP1 was assessed by the “immunoglobulin-coated bead” test, in which the proteins were anchored to beads by specific antibodies. This assay yielded highly reproducible data using both direct titration and displacement approach. The affinities of labeled and unlabeled CTX appeared to be similar for both MMP-2 and NRP1 with estimated *K*_*D*_ values of 0.5 to 0.7 μM. Contrary to previous reports, we found that CTX does not inhibit the activity of MMP-2 and that CTX not only with free carboxyl end but also with carboxamide terminal end binds to NRP1. We conclude that the presented robust assays could also be applied for affinity-improving studies of CTX to its genuine targets using phage display libraries.

Chlorotoxin (CTX) is a small 36 amino-acid cystine-knot miniprotein (1), having a compact tertiary structure stabilized by four disulfide bonds (2). The isolated native CTX as well as its synthetic versions have amidated C terminus, whereas recombinantly produced CTX (hereinafter rCTX) has carboxyl C terminus. While studying glioma-specific chloride currents, it was discovered that CTX exhibits specific and selective binding to glioma cells *in vitro* and *in vivo* as opposed to cells of normal brain tissue. It was also shown that CTX-like molecules with fluorescent or biotin labels can be successfully used for immunohistochemical labeling of gliomas (3). Subsequently, tumor-specific binding by CTX was more broadly observed in various tumors of neuroectodermal origin (4). Lyons *et al.* (4) also demonstrated highly specific binding of biotinylated CTX to immortalized cell lines of different human tumor origins including seven from glioblastomas and six from peripheral neuroectodermal tumors. It was also shown that under physiological conditions, CTX is taken up into tumor and nontumor cells by different internalization processes leading to distinct cellular localization patterns (5). In that study, CTX was proved to be taken up by a receptor-mediated (clathrin-dependent) endocytosis in glioma cells, whereas in normal cells, for example, astrocytes and fibroblasts, CTX was taken up by nonreceptor-mediated macropinocytosis.

Tumor selectivity of CTX was originally attributed to binding to chloride ion channels in glioma cells. Subsequently, matrix metalloproteinase 2 (MMP-2) was identified as a receptor for CTX either alone or in complex with MMP-14 (matrix metalloproteinase 14, also known as membrane type 1-matrix metalloproteinase), tissue inhibitor of metalloproteinase 2 (TIMP-2), and α_v_β_3_ integrin (6). This MMP–TIMP–integrin complex is believed to play a critical role in the regulation (maturation, anchoring, activation, and inhibition) of surface-bound and released active MMP-2, which factors may promote tumor invasion, metastasis, and angiogenesis in concert (7). Binding of CTX to MMP-2 was claimed to be associated with selective inhibition of MMP-2 (6), a desired but difficult to achieve feature among MMP inhibitors because of highly conserved catalytic sites (8).

Nevertheless, in a follow-up study, a direct and specific interaction between Cy5.5-labeled CTX and recombinantly produced MMP-2 could not be detected in a pull-down assay (9). Prompted by this ambiguity, another research group reinvestigated the identity of the glioma CTX receptor and concluded that the direct binding partner protein comprising the CTX receptor is probably annexin A2 (ANX2) (10); although they admitted the possibility of other binding partners and did not verify the CTX–ANX2 interaction in a direct way using recombinantly produced and purified ANX2 protein.

Another research group identified neuropilin 1 (NRP1), an endocytic receptor on tumor and endothelial cells, as a novel CTX target (11). They found that only rCTX binds NRP1; however, the native CTX (*i.e.*, the one with the carboxamide C terminus) does not bind NRP1 but is metabolized to the carboxyl variant by deamidation in cellular environment.

Thus, in view of controversial literature data, the interaction partners(s) of CTX have remained elusive.

Despite the lack of clarity on the binding partners of CTX, because of its tumor-specific binding and cellular uptake, this molecule has attracted much interest in the field of cancer research for utilizations either as diagnostic or therapeutic tool (12). Initially, CTX was proposed as a potential antitumor agent based on either some antiproliferative effects and/or inhibition of MMP-2 and/or inhibition of ANX2 and consequent anti-invasive and antiangiogenic effects. Although some preclinical studies reported effectiveness of synthetically produced CTX on glioblastoma xenograft tumor growth in mice *in vivo* alone or in combination with temozolomide (13, 14), these findings were apparently not confirmed later either by scientific publications or by follow-up clinical studies. Therefore, the interest turned toward utilization of CTX as a specific targeting molecule conjugated with labeling or cytotoxic agents (12).

To obtain conjugated agents, the labeling or payload molecules can be attached to lysine residues of the protein of interest by simple chemical reactions. CTX has three lysine residues at positions 15, 23, and 27. Currently, a monolysine (*i.e.*, K15R, K23R) mutant version of CTX (hereinafter denoted as mCTX) conjugated with indocyanine green, a near infrared fluorophore (mCTX-indocyanine green), denoted as tozuleristide (code name: BLZ-100), is in clinical development as an intraoperative fluorescent imaging agent (https://www.clinicaltrials.gov/study/NCT04743310) (15, 16). Furthermore, CTX is used as the tumor-targeting domain in chimeric antigen receptor T-cell immunotherapy under phase 1 clinical development (https://www.clinicaltrials.gov/study/NCT04214392). The effectiveness of the CTX-chimeric antigen receptor T-cell construct appeared to correlate with MMP-2 expression of the investigated glioblastoma cell lines (17). CTX conjugated with the cytotoxic warhead cryptophycin also appeared to indicate that specific targeting carrier property of CTX can also be utilized for producing peptide–drug conjugates effective *in vivo*. However, the IC_50_ observed with CTX in a transwell migration assay carried out using U251MG, D54MG, and U87MG glioma cell lines was around 600 nM (18), which indicates a moderate potency that might need further optimization to yield higher affinity, functional and targeting potency, and specificity. Our final goal is to discover new CTX analogs that exhibit more potent and specific binding and uptake into glioblastoma and other malignant tumor cells. This is planned to be achieved by phage display biopanning of miniprotein libraries that selects hits based on affinity of CTX analogs to recombinantly produced target protein(s) immobilized on solid surface. For this purpose, taking into consideration the multiplicity of postulated target proteins, we first aimed to establish methods for screening and validating the molecular binding targets of CTX and then to establish an experimental toolset for characterizing target protein binding and functional effects of CTX and CTX-displaying phages and later the new CTX analogs in comparison with wildtype CTX.

## Results

### Molecular toolset for supporting CTX receptor–related studies

**Figure 1:**
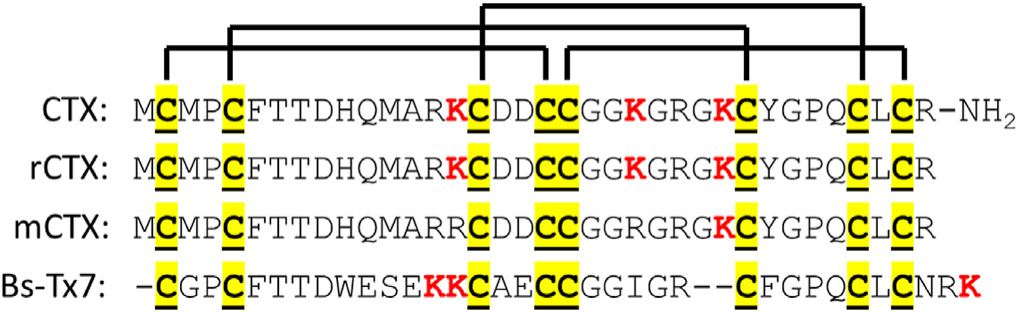
**Amino acid sequences of native and synthetic chlorotoxin having carboxamide C terminus (CTX), recombinantly produced chlorotoxin (rCTX), K27 monolysine mutant chlorotoxin (mCTX), and Bs-Tx7 scorpion toxin.** Disulfide bond pattern is also indicated, and plausible conjugation site lysine (K) residues are highlighted in *red*.

In order to support CTX receptor–binding studies with CTX and its variants, we obtained synthetically produced native CTX with carboxamide C terminus as well as recombinantly produced rCTX and mCTX variants with free carboxyl terminus. For comparison, we also produced recombinant Bs-Tx7, another CTX-like toxin originally isolated from the venom of Indian red scorpion (*Buthus sindicus*) having 66% sequence identity with CTX (19) (Fig. 1). Furthermore, we obtained monoconjugated fluorophore-labeled CTX, rCTX, and mCTX. In most of the studies, cyanine 5-labeled CTX (CTX-Cy5) was used, which emits in the red wavelength range and has high signal to autofluorescence ratio. We also used Alexa Fluor 488–labeled CTX (CTX-A488), which emits in green and has lower signal to autofluorescence ratio.

### Establishing and validation of the immunoglobulin-coated bead test

**Figure 2:**
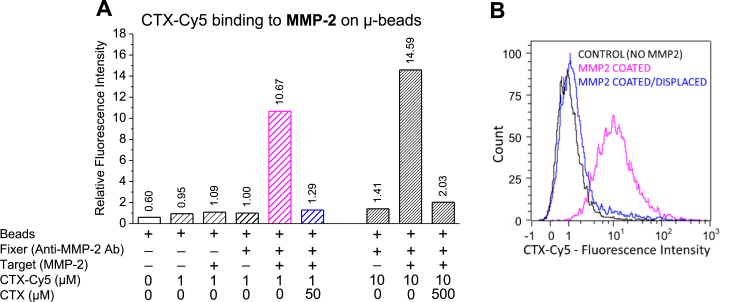
**Quantitative flow cytometric measurement of binding of cyanine 5-labeled CTX (CTX-Cy5) to MMP-2-coated beads.***A*, each column of the bar graph shows relative fluorescent intensities of 10,000 sampled beads subjected to different treatments. The target protein MMP-2 (SRP3118) was anchored to goat anti-rabbit immunoglobulin G-coated beads by rabbit anti-MMP-2 antibody and then the beads were stained with CTX-Cy5. Rare-hatched and densely hatched bars indicate staining with 1 μM and 10 μM CTX-Cy5, respectively. Note that specific high-intensity binding of CTX-Cy5 could be detected only if both the fixer and the target protein were applied before staining. Furthermore, specificity of CTX-Cy5 binding to CTX-binding site(s) could be demonstrated by almost complete displacement of the labeled ligand by pretreatment and coadministration of excess amount of unlabeled ligand. Relative fluorescence intensities were obtained by normalizing median fluorescence intensities (arbitrary intensity values) to that of control beads that were incubated with the fixer but not with the target protein. *B*, intensity distribution histograms of beads treated with 1 μM CTX-Cy5: no target protein control (*black*); MMP-2-coated beads (*magenta*); and MMP2-coated beads treated with excess unlabeled CTX (*blue*). These intensity values are also shown in the fourth, fifth, and sixth columns of the bar graph. MMP-2, matrix metalloproteinase 2.

For investigation of binding affinities of the test substances to selected target proteins, we established a flow cytometry–based binding assay, called “immunoglobulin (Ig)-coated bead test,” in which target proteins are attached to MagnaBind microbeads using protein-specific IgG antibodies. After attaching the target protein, the beads are incubated with a fluorescent ligand, then washed, and the fluorescent intensities of the beads are measured by flow cytometry. We validated the Ig-coated bead test for investigating the binding of CTX-Cy5 to recombinantly produced MMP-2 (Fig. 2). Quantitative evaluation of the fluorescence was based on median fluorescence intensities of beads treated in various ways. It was apparent that uncoated beads or beads coated with only the antibody or the target protein (MMP-2) bound a very small amount of CTX-Cy5 (nonspecific staining) as compared with autofluorescence of beads without staining (Fig. 2). However, the beads coated with both the fixer antibody and MMP-2 showed strong CTX-Cy5 binding–related fluorescent signal indicating specific binding of CTX-Cy5 to MMP-2. To reveal if the binding site of CTX-Cy5 is identical with the binding site of plain CTX, we also investigated displacement of CTX-Cy5 by excess amount of CTX. About 50 μmol per liter CTX almost completely eliminated the binding of 1 μM CTX-Cy5 as indicated either by the bar graph or the histograms of Figure 2, *A* and *B*, respectively.

### Binding of CTX-like ligands to MMP-2 in the Ig-coated bead test

**Figure 3:**
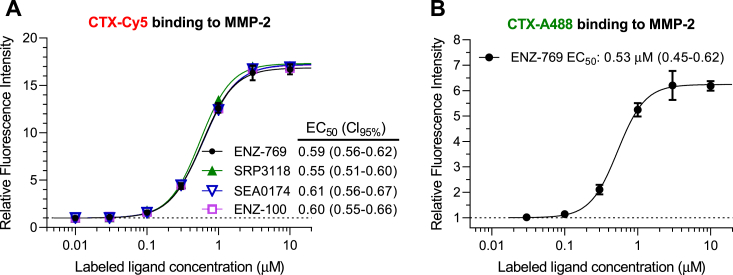
**Concentration dependence of binding of fluorophore-labeled CTX to active and zymogen MMP-2 in the immunoglobulin-coated bead test.***A*, comparison of binding of CTX-Cy5 to four different active (*closed symbols*; ENZ-769 from ProSpec and SRP3118 from Sigma) and zymogen (*open symbols*; SAE0174 from Sigma and ENZ-100 from ProSpec) MMP-2 enzymes. Data for ENZ-769 are presented as mean ± SD of N = 5 with sigmoidal curve fitting. For the other three MMP-2 enzymes, each line and symbols represents one set (concentration series) of seven singlicate measurements. *B*, binding of Alexa 488-labeled CTX (CTX-A488) to a His-tagged active MMP-2 (ENZ-769). Data are presented as mean ± SD of N = 2. The control beads, representing relative fluorescent intensity of 1 (autofluorescence and reflection), were coated with the corresponding MMP-2 protein but were not subjected to staining with labeled CTX. EC_50_ values (with 95% confidence intervals) for each sigmoidal curve fitting are also indicated. Note that the narrow and overlapping confidence intervals indicated that even singlicate or duplicate concentration series were suitable for rather accurately defining the sigmoid concentration-binding curves and EC_50_ values. Differences between EC_50_ values in *A* were not statistically significant. CTX, chlorotoxin; MMP-2, matrix metalloproteinase 2.

**Figure 4:**
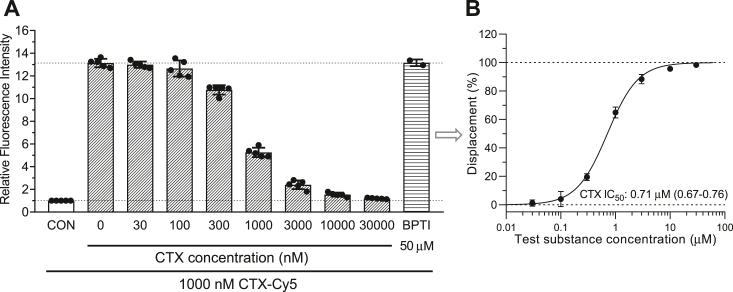
**Concentration-dependent displacement of binding of CTX-Cy5 from MMP-2 by unlabeled CTX in the immunoglobulin-coated bead test.***A*, bar graph of relative fluorescence intensities of bead samples. The control beads (CON) were exposed to staining with 1 μM CTX-Cy5, but MMP-2 was omitted from the coating procedure. As a negative (nonspecific miniprotein) control, the beads shown in the last column were incubated with 50 μM bovine pancreatic trypsin inhibitor (BPTI), another disulfide-rich small protein. *B*, fluorescence intensities at coincubations of the CTX-Cy5-stained beads with increasing concentrations of CTX were converted to a concentration–displacement semilog plot, and IC_50_ value was calculated by sigmoidal curve fitting. Data are presented as means ± SD with superimposed scatter plots in *A* and mean ± SD of N = 5 in *B*. The IC_50_ value with 95% confidence interval in parentheses is also indicated. CTX, chlorotoxin; MMP-2, matrix metalloproteinase 2.

**Figure 5:**
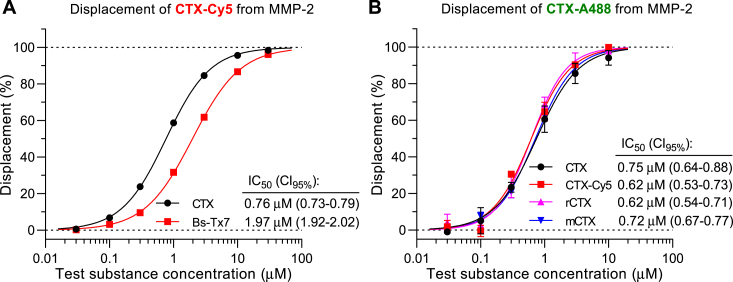
**Comparative assessment of MMP-2 affinities of CTX-like compounds by displacement of fluorophore-labeled CTX from MMP-2 in the immunoglobulin-coated bead test.***A*, comparison of affinities of CTX and the *Buthus sindicus* scorpion venom toxin Bs-Tx7 by concentration–displacement curves against 1 μM CTX-Cy5. Both lines in the graph represent one set of singlicate measurements. Difference between the two IC_50_ values was statistically significant (*p* < 0.0001). *B*, comparison of affinities of CTX, mCTX, rCTX, and CTX-Cy5 by concentration–displacement curves against 1 μM CTX-A488. Data are presented as mean ± SD of two experiments for each test substance. Differences between the four IC_50_ values were not statistically significant. IC_50_ values with confidence intervals are also indicated in *A* and *B*. CTX, chlorotoxin; mCTX, mutant CTX; MMP-2, matrix metalloproteinase 2; rCTX, recombinantly produced CTX.

The Ig-coated bead test was used for investigating affinities of ligands in two different test modes, that is, direct binding and competitive displacement. For assessing affinities of fluorophore-labeled ligands to CTX receptors, the direct assay was applied, in which the amount of bound ligand was estimated directly from fluorescence intensities after incubation with various concentrations of the labeled ligand (Fig. 3). For assessing affinities of unlabeled ligands to CTX receptors, the competitive assay was applied, in which the beads were treated with a fixed concentration of a labeled ligand (1 μM CTX-Cy5) after preincubation and in coincubation with the tested unlabeled ligand, and the bead-bound fluorescence intensity was measured (Fig. 4). Relative affinities of unlabeled ligands were estimated by concentration–displacement studies characterizing the 50% displacing concentrations (IC_50_ values) of the test ligands. In the direct binding study, we compared concentration–intensity relationships for CTX-Cy5 binding to the zymogen pro-MMP-2 and to the mature active MMP-2, with both variants obtained from two vendors (Fig. 3*A*). In the figure, we show concentration–intensity relationships on an active MMP-2 (ENZ-769) as pooled results of five experiments (N = 5) with a series of ligand concentrations, whereas the binding to the other three MMP-2 preparations is presented as fitted curves on intensities plotted against a seven-point concentration series (“singlicate experiments”). The results showed practically identical concentration–intensity relationships for CTX-Cy5 binding to both the zymogen and active MMP-2 and to all the four MMP-2 preparations with points fitting well to a sigmoidal curve. The concentration–intensity curves reached saturation at the same intensity levels with both enzyme versions suggesting equal number of binding sites on beads coated with either type of MMP-2. The potencies reflected by half-maximal intensities (EC_50_ values between 0.55 and 0.61 μM with overlapping confidence intervals) were also practically identical, suggesting equal affinities to the two types of enzymes. The low variability of repeated experiments and the good fitting indicated excellent robustness of the test. As the 95% confidence intervals for the seven-point singlicate EC_50_ determinations were only slightly wider (approximately ± 10%) than that of five pooled experiments (±5%), we concluded that even singlicate or duplicate experiments might be sufficient for determination of the labeled ligand affinities with the essential precision. Similar conclusion could be drawn for IC_50_ determinations in the competitive displacement experiments (see data variability in Fig. 4). For comparative characterization of affinities of red fluorescence–labeled (CTX-Cy5) and unlabeled CTX ligands in displacement test, we have also established the binding assay with a green fluorophore–labeled CTX, CTX-A488. The binding curve for CTX-A488 (Fig. 3*B*) was very similar to that for CTX-Cy5 with good sigmoidal fit and similar EC_50_ value (0.53 μM) but lower E_max_ in terms of relative fluorescence intensity (RFI). Using 1 μM CTX-Cy5 as a displaced ligand, we compared concentration–displacement relationships for unlabeled CTX and Bs-Tx7. Both toxins caused almost complete displacement of the labeled ligand at the highest concentration used (30 μM) with good sigmoidal fit and apparent parallel concentration–displacement curves suggesting that they have a common binding site with CTX-Cy5 (Fig. 5*A*). Thus, the competitive displacement test on Ig-coated beads appeared to be suitable for assessing relative affinities of CTX-like compounds to MMP-2. Bs-Tx7 appears to bind to MMP-2 in a similar manner, but with 2.6-fold lower affinity than CTX, as concluded from the ratio of their IC_50_ values. Using 1 μM CTX-A488 as displaced ligand, we compared relative affinities of CTX, CTX-Cy5, rCTX, and mCTX in the CTX-A488 displacement test (Fig. 5*B*). The results indicated almost the same affinity for all four test substances. Most importantly, affinity of CTX-Cy5 was similar to that of unlabeled CTX.

### Effect of CTX on enzymatic activity of recombinantly produced MMP-2

**Figure 6:**
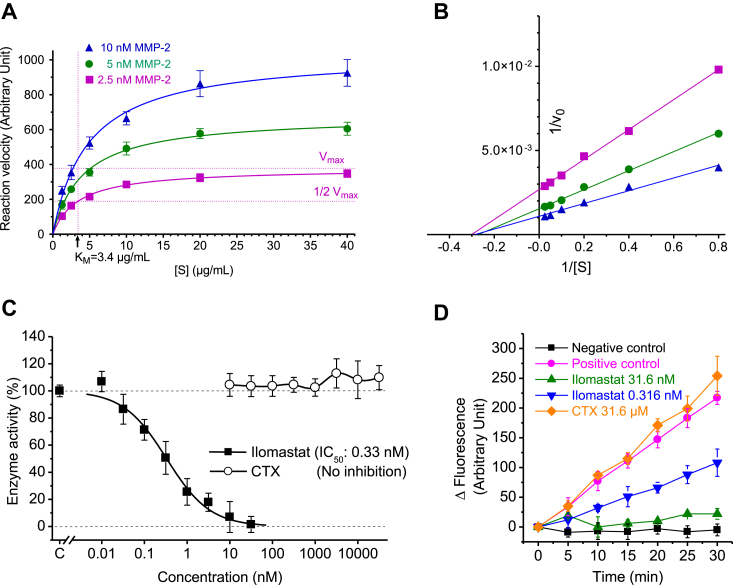
**Lack of effect of CTX on enzymatic activity of MMP-2.** Michaelis–Menten (*A*) and Lineweaver–Burk plots (*B*) from the enzyme kinetic experiment (N = 4, V_0_ mean ± SD) to establish assay conditions for the gelatin degradation assay with recombinant active MMP-2 enzyme and DQ-gelatin as cleavage indicator substrate (S). *C*, effects of CTX and ilomastat (positive control for MMP inhibition) on gelatinolytic activity of MMP-2. Data are presented as mean ± SD. For the controls, each point represents the mean of eight measurements. For ilomastat and CTX, each point represents five and four measurements, respectively. For CTX groups, the difference from control was not statistically significant (*p* = 0.34, one-way ANOVA). *D*, representative time course of fluorescence plots from the experiments shown in *C*. Data are presented as mean ± SD of change in fluorescence (ΔFluorescence). CTX, chlorotoxin; DQ gelatin, fluorogenic dye–quenched gelatin; MMP-2, matrix metalloproteinase 2.

**Figure 7:**
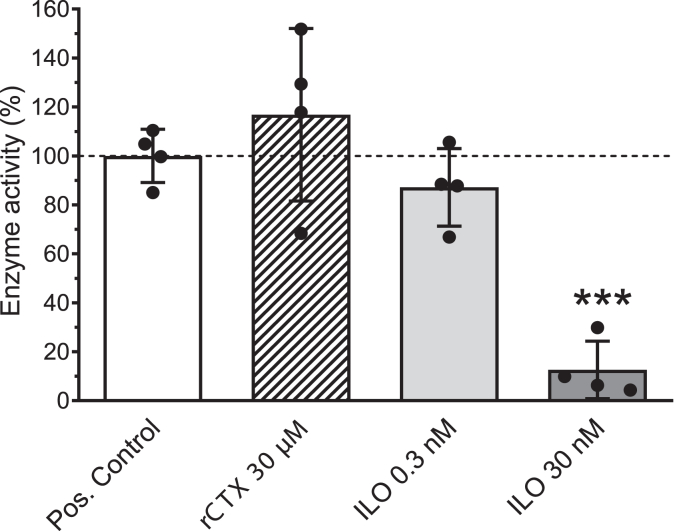
**Lack of effect of rCTX (30 μM) on enzymatic activity of pro-MMP-2 activated by APMA.** A low and a high concentration of ilomastat was also tested as inhibitor positive control. Enzyme activity was detected as the increase in fluorescence because of cleavage of the peptide substrate FS-6 (50 μM). Data are presented as mean ± SD of quadruplicate measurements with superimposed scatter plot. ∗∗∗ indicates statistically significant difference (*p* < 0.001) from positive control (ANOVA followed by Dunnett’s test). APMA, p-amino-phenylmercuric acetate; MMP-2, matrix metalloproteinase 2; rCTX, recombinantly produced CTX.

To investigate the functional consequence of binding of CTX to MMP-2, we tested the effect of CTX on the enzymatic activity of recombinantly produced active form of MMP-2. The assay was performed using fluorogenic dye–quenched (DQ) gelatin (DQ gelatin) as indicator substrate, and the slope of the change in fluorescent intensity indicated the velocity of enzymatic cleavage of the substrate. Prior to testing enzyme inhibition, the time course of the reaction and initial velocities (V_0_) were determined at various substrate concentrations and three different enzyme concentrations, from which the maximum velocity (V_max_) and the Michaelis constant (*K*_*M*_) values were determined (Fig. 6, *A* and *B*). Based on this experiment, the enzyme concentration of 2.5 nM and the substrate concentration of 3.5 μg/ml were selected for testing the inhibitory effect of CTX. CTX was tested in a broad concentration range from 10 nM to 30 μM in parallel with an adequate concentration series of ilomastat used as positive control. CTX had no statistically and biologically significant effect on MMP-2 activity, whereas ilomastat caused concentration-dependent inhibition as expected (Fig. 6, *C* and *D*). We also tested the inhibition of MMP-2 activity by CTX using a fluorescence-quenched oligopeptide substrate FS-6 (20) and recombinant pro-MMP-2 enzyme activated by p-amino-phenylmercuric acetate (APMA). We applied rCTX at a high concentration (30 μM) along with ilomastat as positive control. However, this test also failed to indicate any significant effect of rCTX on the enzymatic activity of MMP-2 (Fig. 7).

### Establishing the cobalt-coated bead test

In order to investigate which other postulated target proteins could be identified as direct binding partners of CTX, we established another microbead- and flow cytometry–based assay, in which His-tagged target proteins are immobilized on the surface of cobalt-coated beads and applied this method for testing binding intensity of CTX-Cy5 to various target proteins. Furthermore, the cobalt-coated bead test was also customized for investigation of binding of various protein-displaying phages to different immobilized target proteins.

### Investigation of CTX binding to a panel of potential target proteins

**Figure 8:**
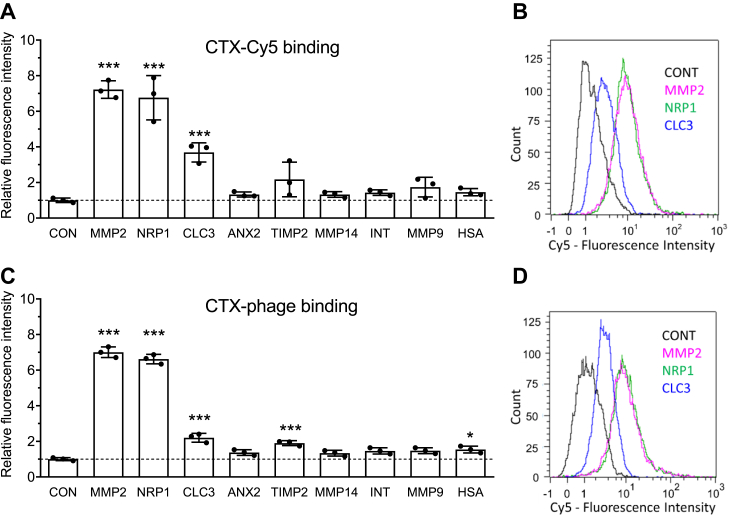
**Binding of CTX-Cy5 and CTX-displaying phages to various proposed target proteins and human serum albumin in the cobalt-coated bead test.** “His-tag isolation and pulldown” Dynabeads were coated with various His-tagged proteins at 0.16 μM for each. The tested target proteins were MMP-2, NRP1, CLC-3 chloride channel, TIMP-2, MT1-MMP (MMP14), annexin A2 (ANX2), α_v_β_3_ integrin (INT), MMP-9, and human serum albumin (HSA). *A*, the coated beads were blocked with casein and stained with 1 μM CTX-Cy5. Control beads (CONT) were not incubated with any target protein but blocked with casein and stained. *B*, representative intensity distribution histograms of beads from an experiment of *A*. *C*, the coated beads were blocked with casein, incubated with CTX-displaying phages (5.5 × 10^14^ particles/ml), and stained to detect M13 phage binding. Control beads (CONT) were not incubated with any target protein but blocked with casein, and phages were also applied and stained. The bar graphs in *A* and *C* represent relative fluorescence intensities calculated from median fluorescence intensities of 10,000 events recorded by the flow cytometer and are presented as mean ± SD of three separate experiments with superimposed scatter plot. ∗ and ∗∗∗ indicate statistically significant difference (*p* < 0.05 and *p* < 0.001) from control (ANOVA followed by Dunnett’s test). *D*, representative intensity distribution histograms of beads from the experiment of *C*. Note the very similar patterns of binding of fluorescent-labeled and phage-displayed CTX to various target proteins. CTX, chlorotoxin; MMP-2, matrix metalloproteinase 2; NRP1, neuropilin 1; TIMP-2, tissue inhibitor of metalloproteinase 2.

We investigated the binding of 1 μM CTX-Cy5 to the following panel of His-tagged target proteins in the cobalt-coated bead test: MMP-2, NRP1, MMP-9, TIMP-2, MMP-14, ANX2, α_v_β_3_ integrin, CLC-3 chloride channel, and also human serum albumin as a nonspecific protein control. The results showed that labeled CTX bound not only to MMP-2 but also to NRP1 with similar intensity (Fig. 8*A*). In addition, CLC-3 and TIMP-2 appeared to exhibit weaker but appreciable binding of the labeled CTX. We also investigated the binding of CTX-displaying phages to the same panel of proteins in the cobalt-coated bead test (Fig. 8*B*). Interestingly, the binding pattern of CTX-displaying phages was very similar to that seen with labeled CTX, that is, similarly strong binding to MMP-2 and NRP1 and weaker but appreciable binding to CLC-3 and TIMP-2, compared with the nonspecific binding level represented by human serum albumin and the other proteins tested.

### Characterization of NRP1 binding in Ig-coated bead test

**Figure 9:**
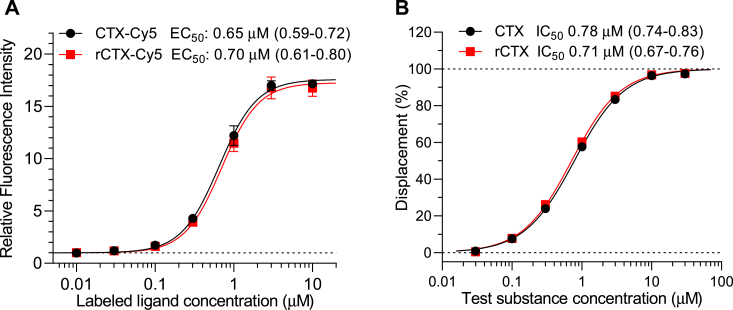
**Characterization of binding affinity of synthetic and recombinant CTX variants to NRP1 in the immunoglobulin-coated bead test.***A*, binding of the labeled ligands CTX-Cy5 and rCTX-Cy5. *B*, displacement of CTX-Cy5 (1 μM) by unlabeled CTX. Data are presented as mean ± SD of two independent experiments in *A* and as calculated percent displacements at a series of concentrations from singlicate measurements in *B*. IC_50_ values with confidence intervals are also indicated in *A* and *B*. Difference between the compared IC_50_ values was not statistically significant in *A* but was statistically significant in *B* (*p* = 0.019), though technically insignificant. CTX, chlorotoxin; NRP1, neuropilin 1; rCTX, recombinantly produced CTX.

As the binding of labeled CTX to NRP1 was almost as strong as to MMP-2, we also aimed at characterizing the affinity of this binding in the Ig-coated bead test. Since proponents of NRP1 as CTX receptor claimed a difference between binding of rCTX and C-terminally amidated CTX to NRP1, we also compared the NRP1-binding potencies of these two CTX variants both in labeled and unlabeled forms. Like in the case of MMP-2 binding, NRP1 binding of both labeled CTX variants also showed sigmoidal saturation binding curve. In contrast with the findings of McGonigle *et al.* (11), both CTX-Cy5 and rCTX-Cy5 were found to bind to NRP1 with practically equal affinities as indicated also by EC_50_ values 0.65 μM and 0.70 μM, respectively (Fig. 9*A*). The displacement study against 1 μM CTX-Cy5 also showed well-fitted sigmoidal displacement curves for both CTX and rCTX with technically identical affinities as indicated by IC_50_ values of 0.78 μM and 0.71 μM, respectively (Fig. 9*B*). These results also indicated that affinities of Cy5-labeled and unlabeled CTX to MMP-2 and NRP1 are similar.

**Figure 10:**
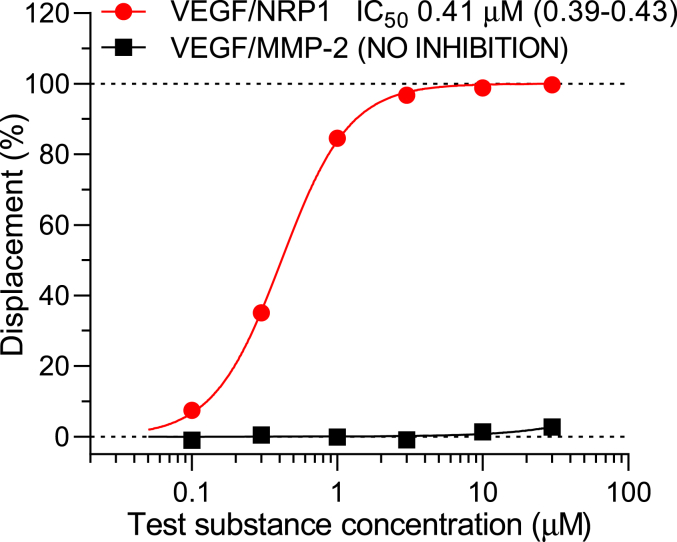
**VEGF-A165 concentration-dependently displaces CTX-Cy5 (1 μM) from NRP1 but not from MMP-2-coated beads (protein-specific immunoglobulin-coated bead).** Data are presented as calculated percent displacements at a series of concentrations from singlicate measurements. CTX, chlorotoxin; MMP-2, matrix metalloproteinase 2; NRP1, neuropilin 1; VEGF, vascular endothelial growth factor.

Since NRP1 is a receptor for vascular endothelial growth factor (VEGF) family members and binds VEGF-A165 with high affinity (21), we investigated if it could displace 1 μM CTX-Cy5 from NRP1. For comparison, we also tested CTX-Cy5 displacement from the MMP-2 protein–coated beads (Fig. 10). The results showed that VEGF-A165 could completely abolish CTX-Cy5 binding to NRP1 with a sigmoidal concentration–inhibition relationship, which is steeper than that for CTX (*cf.* Fig. 9*B*; Hill slopes [confidence intervals]: 1.86 [1.73–2.01] *versus* 1.22 [1.15–1.29]), whereas it did not affect at all labeled CTX binding to MMP-2. These results also showed higher displacing potency of VEGF-A165 from the CTX receptor on NRP1 (*i.e.*, lower IC_50_ = 0.41 μM) as compared with that of CTX (IC_50_ = 0.78 μM).

### Validation of the cobalt-coated bead test for characterizing binding strength of phage-displayed protein ligands to CTX receptors on MMP-2 and NRP1

**Figure 11:**
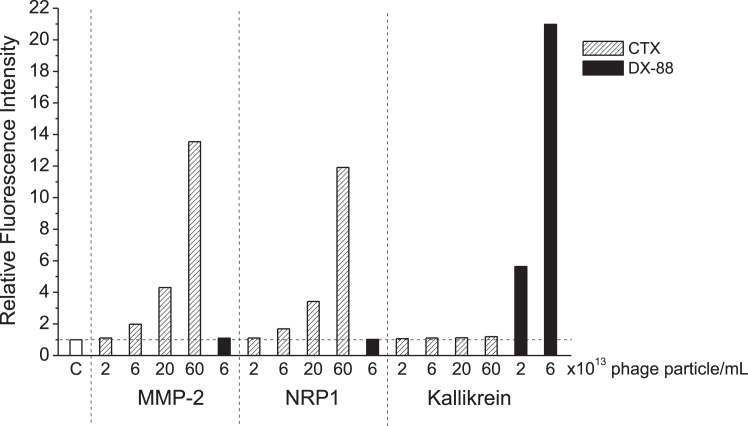
**Concentration-dependent binding of CTX- and DX-88-displaying phages to MMP-2, NRP1, and kallikrein in the cobalt-coated bead test.** The His-tagged target proteins were anchored on the surface of the cobalt-coated beads and blocked with casein. These beads were exposed to CTX- or DX-88-displaying phage solutions at various phage concentrations. Control beads (C) were not incubated with any target protein but blocked with casein; no phages were applied, only staining for attached phages was performed. Therefore, relative fluorescence intensity (RFI) values above 1 (ΔRFI = RFI – 1) represent the phage binding–related signals. Note that the nonspecific binding of highest concentrations of phages to mismatching proteins, that is, 6 × 10^13^ particle/ml DX-88 phage to MMP-2 and NRP1 or 6 × 10^14^ particle/ml CTX phage to kallikrein was very low, ΔRFI = 0.10, 0.04, and 0.20, respectively. CTX, chlorotoxin; MMP-2, matrix metalloproteinase 2; NRP1, neuropilin 1.

Since we are aiming to develop new potent CTX analogs by biopanning a miniprotein library by phage display technology, we need a hit confirmation test enabling investigation of binding strength of phage-displayed CTX analogs as compared with phage-displayed CTX. Therefore, we produced a CTX-displaying phage clone, customized, and validated the flow cytometry–based cobalt-coated bead test for quantitative assessment of the binding tendency of ligand-displaying phages to target proteins immobilized on the surface of microbeads. To support the validation and to investigate the specificity of binding, we also produced a phage clone that displays ecallantide (DX-88), which is an engineered small protein inhibitor of kallikrein. DX-88 was one of the first protein drugs identified through phage display technology (22). A validation experiment demonstrating apparent intensity of specific and nonspecific binding of protein-displaying phages in the cobalt-coated bead test is shown in Figure 11. For validation, and to show liability of the test to detect specific and nonspecific binding, we added different concentrations of CTX- and DX-88-displaying phages onto the beads coated with “matching” and “mismatching” target proteins, followed by appropriate washing (Fig. 11). For DX-88 phages, the “matching” target protein was kallikrein, whereas for CTX phages, it was MMP-2 and NRP1. The measured fluorescence intensity was clearly dependent on phage concentration for matching ligand and target proteins. In contrast, the nonspecific binding of phages at mismatching ligand–phage to target protein pairs was negligible up to the highest phage concentrations that provided large intensity signals on matching target protein coatings, that is, 6 × 10^14^ particle/ml for CTX phages and 6 × 10^13^ particle/ml for DX-88 phages. The phage concentrations needed for an appropriate signal were much lower for DX-88–kallikrein pair with subnanomolar affinity (23), than for CTX having submicromolar EC_50_/IC_50_ values at binding to its partner proteins MMP-2 and NRP1.

## Discussion

The scientific literature strongly suggested that CTX might be a specific targeting molecule for recognizing gliomas and various other malignant tumor cells. Thereby, it may serve anticancer diagnostic and therapeutic purposes (12). However, CTX had relatively moderate potency with effective concentrations in the submicromolar range. For example, the IC_50_ of CTX in a transwell migration assay with glioma cells was about 600 nM (18). Therefore, it might be worth to further optimize CTX to obtain new derivatives with higher affinity and specificity, first to its relevant target protein, and ultimately to cancer cells. Phage display is a powerful method for affinity-enhancing optimization of proteins/peptides. As an initial step for phage-display–based optimization, first we aimed to identify the genuine target protein(s) of CTX that would enable solid phase biopanning of CTX-displaying phages. The number one candidate was MMP-2, which was identified by Deshane *et al.* (6) as CTX receptor on glioma cell membranes by affinity purification followed by mass spectroscopy and sequencing. Using an ELISA-based method, they have also demonstrated binding of His-tagged rCTX to recombinantly produced MMP-2. However, the method was not specified in detail in their publication. Nevertheless, subsequently, Veiseh *et al.* (9) failed to confirm the specific binding of fluorophore- (cyanine 5.5) labeled CTX to recombinantly produced and purified MMP-2 in a pull-down experiment. These ambiguous results prompted us to invent new and reliable methods for verifying or disproving the direct and specific binding of CTX to MMP-2 or other potential target proteins and also for effective characterization of affinities of CTX-like ligands. Additional requirements were that the assays to be developed should be robust, require relatively small amounts of commercially available target proteins immobilized on solid surface, characterized by low levels of nonspecific binding, and also applicable for testing CTX analog–displaying phages. Based on the aforementioned, our primary objective in this work was the establishment and validation of two flow cytometry– and fluorescently labeled CTX-based methods, the Ig-coated bead test that uses specific antibodies for anchoring target proteins, and the cobalt-coated bead test, which utilizes the high affinity between metal ions and His tags. The representative validation experiment for the Ig-coated bead test (Fig. 2) demonstrated that (1) the fluorophore-labeled CTX exhibited low nonspecific binding when the beads were uncoated with the matching target protein; (2) high specific binding was observed when all conditions for appropriate target protein coating were fulfilled; and (3) the fluorescent signal provided by the bead-bound fluorophore–labeled CTX represented indeed a binding to a CTX-binding site, since the labeled ligand could be almost completely displaced by unlabeled CTX. It appears that the Ig-coated bead test provides higher accuracy associated with low nonspecific binding in comparison with traditional ELISA tests, which might be critical in the case of moderate affinity ligands, such as CTX.

Both our direct binding assays with labeled CTX and the competitive displacement studies showed well-fitting sigmoidal curves in semilog plots, further strengthening the notion that MMP-2, either active- or pro-MMP2, contains a binding site for CTX, for which a *K*_*D*_ value of 0.5 to 0.6 μM can be estimated. The results also demonstrated that our Ig-coated bead test is suitable for assessing relative affinities of CTX analog compounds, and the Cy5-labeled CTX, unlabeled CTX, rCTX, and mCTX all have very similar affinities. To the best of our knowledge, this is the first direct confirmation of CTX binding to recombinantly produced and purified MMP-2 protein. Our results confirm that MMP-2 is indeed a target protein of CTX, based on which CTX analogs might be optimized by phage display technology for higher affinity and specificity to further improve specific targeting of tumor cells.

It has previously been claimed that the binding of CTX was associated with inhibition of enzyme activity of MMP-2 (6, 24). The results presented in both publications showed approximately 40% inhibition of the enzyme activity at a concentration of 3 μM CTX, which appeared to be a maximum achievable effect based on the concentration–inhibition curve shown by Deshane *et al.* (6). On the contrary, we found no inhibition of MMP-2 activity by CTX even at a concentration of 30 μM either using a recombinantly produced active MMP-2 or APMA-activated pro-MMP-2 preparations, whereas the activity of the enzymes and sensitivity of the assays were confirmed by including appropriate controls. Furthermore, both CTX and rCTX were devoid of any inhibitory effects on MMP-2. We do not know the reason for the discrepancy between the previously published findings and our results, particularly because those methods were not specified in detail, just referring to instructions of test kits (from Chemicon, later Millipore), which appear to be unavailable today. However, from the limited description mentioning the use of biotinylated gelatin– and biotin-binding plates, it may be assumed that the applied methods in those articles were similar to that described by Ratnikov *et al.* (25). This assay detects the cleavage of biotinylated gelatin by binding the cleaved fragments to a streptavidin-coated microtiter plate, which is then washed and stained. The signal is developed in an ELISA-like process. This is a quite complex process based on endpoint measurements giving rise to numerous potential artifacts. In contrast, our primary method using DQ gelatin converts the gelatin cleavage to fluorescent signal directly in an apparently linear manner, the kinetics of which process can be followed in a plate reader. Besides comparability to the mentioned publications, the use of DQ gelatin as primary indicator substrate was also justified by that CTX was proposed to bind to the collagen-binding domain of MMP-2, which is also the binding site of gelatin (26).

Besides MMP-2, there are other potential target proteins of CTX. It was described that MMP-2 persists on the cell surface as a protein complex involving also MMP-14, TIMP-2, and α_v_β_3_ integrin (6). The chloride channel inhibitory effect of CTX on glioblastoma cells (18) was attributed to blockade of CLC-3 chloride channels (27, 28). However, it was also proposed that CTX does not directly inhibit CLC-3 channels but depletes them from the cell surface by internalization into caveolar rafts (28) as MMP-2 and CLC-3 colocalize in membrane domains (29), or CLC-3 participates in the protein complex involving MMP-2 and MMP-14 (30), and CTX binding to MMP-2 facilitates the internalization. ANX2 was proposed as an alternative binding target of CTX instead of MMP-2 (10). More recently, NRP1 was suggested as the binding partner for CTX with the caveat that only deamidated CTX (*i.e.*, rCTX) but not the native CTX with carboxamide C terminus binds to NRP1 (11). The results described previously prompted us to establish a reliable comparative binding method, the so-called cobalt-coated bead assay. It enables immobilization of various recombinantly produced and purified protein preparations to microbeads in a uniform manner utilizing high metal ion affinity of N- or C-terminal His tags. With this, one can investigate a panel of proteins that were suspected to bind to CTX. Besides the previously proposed target proteins, that is, MMP-2, ANX2, NRP1, and CLC3, we also included those in the tested panel of proteins, which were reported to participate in the MMP–TIMP–integrin complexes that bind CTX (6). Furthermore, we added MMP-9, the closest relative of MMP-2, which reportedly does not bind to CTX (6), and human serum albumin as nonspecific binding control. It was also interesting to compare the target protein binding profile obtained with Cy5-labeled CTX- and the one with CTX-displaying phages, which were highly concordant.

According to our results, labeled CTX- or CTX-displaying phages bound to NRP1 with similar binding affinity as to MMP-2. However, this finding contradicted with the previous results that only rCTX but not CTX binds to NRP1 (11), since we used CTX-Cy5 and not rCTX-Cy5 in this study. Moreover, our phage-displayed CTX did not have free carboxy C terminus, as it was fused to the N terminus of the P3 coat protein *via* a GSASSATR linker. We further characterized NRP1 binding of CTX in the Ig-coated bead assay to compare the affinity of this interaction with labeled and unlabeled CTX and rCTX. These comparative binding studies consistently indicated practically equal affinities for the two variants. In line with the protein panel study with the cobalt-coated bead test, these experiments indicated slightly lower affinity of CTX to NRP1 compared with MMP-2. CTX-Cy5 could be effectively displaced from NRP1 but not from MMP-2 by VEGF-A165. These findings also point to specificity of these displacement studies and suggest that the binding site of CTX on NRP1 overlaps with the binding site of VEGF-A165. We do not know the reason for the discrepancy between our results and those of McGonigle *et al.* (11) concerning the binding or lack of binding of native CTX to NRP1, but we can note crucial differences between the two methods. In our Ig-coated bead test, NRP1 was immobilized on solid surface, and labeled or unlabeled CTX or rCTX was in solution. In the biolayer interferometry assay of McGonigle *et al.* (11), biotinylated CTX was immobilized on the solid surface of a biosensor chip, and NRP1 was in solution. Both samples comprised a1, a2, b1, and b2 extracellular domains of NRP1 (31) with the difference that in our bead tests, NRP1 was fused to a His tag, whereas in the biolayer interferometry study to the Fc region of human IgG1 at the C terminus. Modeling and crystallization studies are in progress in our laboratory to address this discrepancy and to learn structural details how CTX variants interact with both NRP1 and MMP-2.

In addition to MMP-2 and NRP1, CLC-3 and TIMP-2 appeared to exhibit weaker but appreciable binding to labeled and phage-displayed CTX. Our finding raises the possibility of a direct interaction between CTX and CLC-3 in line with the first identification of CTX as a direct chloride channel blocker demonstrated by single-channel recordings (32), which cannot be explained by endocytotic internalization. The finding concerning TIMP-2 suggests the possibility that the MMP–TIMP–integrin complex could be a cell surface receptor for CTX, and the weaker binding site on TIMP-2 may contribute to the binding of CTX.

Our study could not confirm ANX2 as a direct CTX receptor. The proposal of ANX2 being a CTX receptor was based on antiangiogenic effects of CTX including functional antagonism against effects of VEGF and that knockdown of ANX2 was associated with depletion of cell surface CTX-binding sites (10). However, the antiangiogenic effects might also be attributed to the competition of CTX at the VEGF coreceptor on NRP1. Moreover, since ANX2 is a pleiotropic protein involved in multiple cellular functions, including endocytosis, exocytosis, membrane domain organization, protein assembly, and transcription (33), the CTX receptor depletion could also be a secondary effect. Nevertheless, ANX2 is subject to complex regulation by post-translational modifications, most importantly phosphorylation at numerous sites affecting its cellular functions (33). In this study, we used ANX2 that was recombinantly produced in *Escherichia coli*. Therefore, if any post-translational modification were needed to switch on ANX2 as CTX receptor, then the lack of ANX2 binding to CTX receptor might be due to the lack of such modification. Finally, it is also notable that biochemical identification of ANX2 as a target for CTX was based on crosslinking, pull-down, and mass spectrometry analysis. Therefore, it is also conceivable that the authors identified ANX2 as not a direct interaction partner but a component of a complex containing a genuine CTX target (either NRP1 or MMP-2).

In general, it was not only a virtue but also a limitation of the present target identification study that we investigated binding of CTX to recombinantly produced proteins attached to solid surface but not in their physiological cellular and molecular environments. Some of the proteins were produced in bacteria and did not undergo post-translational modifications that might alter their binding properties. Theoretically, it is also a limitation that the target scanning was carried out with labeled CTX. If the labeling prevented the protein–protein interaction between CTX and a tested protein, then that interaction might have remained unrevealed. However, the similar pattern of relative binding intensities observed with phage-displayed as well as labeled CTX at least attenuated the risk of overlooking a potential target because of interference of the fluorophore at the conjugation site with binding of CTX. Our validation experiment on the utility of cobalt-coated bead test to specifically detect binding of phage-displayed proteins (or peptides) to target proteins immobilized on these beads with excellent specific to nonspecific binding ratio demonstrated the usefulness of this novel assay for target protein screening as well as confirmation of hits emerging from biopanning of phage display libraries, even including proteins with moderate affinity, like that of CTX.

In conclusion, two microbead-based assays were established and validated for direct target protein identification and affinity characterization of CTX and similar miniproteins. Our interaction partner identification study confirmed two suggested but previously debated or unconfirmed target proteins of CTX, that is, MMP-2 and NRP1. On the other hand, we could not corroborate the interaction of CTX with ANX2. Moreover, contrary to previous reports, we found that CTX does not inhibit the gelatinolytic activity of MMP-2, and not only recombinant CTX with free carboxyl terminal but also synthetic CTX with carboxamide terminal binds to NRP1. Finally, a weak binding was also observed to purified CLC-3 chloride channel and TIMP-2. Our results clearly showed that MMP-2 and NRP1 are genuine binding partners of CTX, at least in a cell-free system. Whether they are genuine targets of CTX *in vivo*, it needs further validation experiments using cancer cell lines.

## Experimental procedures

### Unlabeled CTX-related test substances

Synthetically produced CTX was purchased from Iris Biotech.

For the recombinant production of rCTX, mCTX, and Bs-Tx7, the corresponding DNA sequence was cloned into a pET-based expression vector (34) as C-terminal fusion to DsbC (disulfide bond isomerase C) and the recognition site Trp-Glu-Leu-Gln of the staphylococcal serine protease SplB. The DsbC–CTX fusion proteins were produced in the cytoplasm of SHuffle T7 Express cells (New England Biolabs) grown in autoinduction terrific broth at 30 °C for 30 h.

The cells were harvested, resuspended in 50 mM Tris–HCl (pH 7.5), 300 mM NaCl, and 0.5% Triton X-100, and disrupted by sonication. The cell debris was pelleted by centrifugation at 48,400*g*. The His-tagged DsbC–CTX and DsbC–Bs-Tx7 fusion proteins were isolated from the cytoplasmic fraction using immobilized metal ion affinity chromatography (IMAC). The column was equilibrated and washed with 50 mM Tris–HCl (pH 7.5), 300 mM NaCl, and 30 mM imidazole, and the protein was eluted with 250 mM imidazole.

From this point, purification of rCTX, mCTX, and Bs-Tx7 was carried out in a slightly different manner. The eluate containing recombinant CTX was dialyzed against 20 mM Tris–HCl (pH 8.0) buffer overnight at 4 °C. The dialyzed sample was applied to a HiTrap Q HP column (GE Healthcare) equilibrated with 20 mM Tris–HCl (pH 8.0) buffer, and the fusion protein was eluted with a 0 to 50% linear gradient 1 M NaCl. The DsbC–CTX containing fractions were pooled. To cleave CTX off its fusion partner, the sample was supplemented with His-tagged SplB protease (produced recombinantly in *E. coli* using an expression construct that was a kind gift of Bence Kiss) and reducing agent Tris(2-carboxyethyl) phosphine (TCEP) at a 3:1:1 fusion protein:protease:TCEP molar ratio and incubated overnight at 25 °C. The sample was again subjected to IMAC to remove the His-tagged DsbC and SplB from the sample. The column was washed with 20 mM Tris–HCl (pH 8.0), 0.5 M NaCl, and 15 mM imidazole buffer. The flow-through containing rCTX was collected and concentrated using an Amicon Ultra-15 centrifugal filter unit (Merck Millipore Ltd; molecular weight [MW] cutoff [MWCO] = 3000 Da). At the same time, the buffer was exchanged to 10 mM Tris–HCl (pH 8.0) and 0.5 M NaCl for the subsequent size-exclusion chromatography step. The sample was applied to a HiLoad 16/600 Superdex 30 pg column (GE Healthcare), and rCTX was eluted with one column volume of buffer. The Tris–HCl and NaCl contents of the rCTX-containing fraction were reduced by repeatedly concentrating the sample on an Amicon Ultra-15 centrifugal filter unit (MWCO = 3000 Da) and diluting it in US Pharmacopeia–tested water for injection. The purity and molecular mass of the protein were verified by subsequent HPLC–UV/MS measurements (Fig. S1).

The DsbC–mCTX fusion protein was dialyzed against 20 mM Hepes (pH 7.5). After dialysis, SplB and TCEP were added in a 3:1:1 fusion protein:protease:TCEP molar ratio, and the sample was incubated overnight at 25 °C. The sample was subjected to cation exchange chromatography to get rid of contaminating proteins flowing through the column. The sample was applied to a HiPrep SP HP column (GE Healthcare) equilibrated with 20 mM Hepes (pH 7.5) buffer, and mCTX was eluted in a single step with 20 mM Hepes (pH 7.5) and 600 mM NaCl. The eluate was concentrated using a Pall Microsep Advance centrifugal filter unit (MWCO = 1000 Da). mCTX was further purified using size-exclusion chromatography on a HiLoad 16/600 Superdex 30 pg column equilibrated with 20 mM Hepes (pH 7.5) and 0.15 M NaCl buffer. The buffer and salt concentration of the mCTX-containing fraction was reduced by repeatedly concentrating the sample on a Pall Microsep Advance centrifugal filter unit (MWCO = 1000 Da) and diluting it in US Pharmacopeia–tested water for injection. The purity and molecular mass of the protein were verified by subsequent HPLC–UV/MS measurements (Fig. S2).

The DsbC–Bs-Tx7 fusion protein was dialyzed against buffer containing 50 mM Na_2_HPO_4_ (pH 7.0). After dialysis, SplB and TCEP were added in a 3:1:1 fusion protein:protease:TCEP molar ratio, and the sample was incubated overnight at 25 °C. The pH of the sample was adjusted to 4.2 with citric acid, and the sample was subjected to cation exchange chromatography. The sample was purified on a HiPrep SP HP column equilibrated with 50 mM Na_2_HPO_4_ (pH 4.2) buffer, and Bs-Tx7 was eluted in a single step with 50 mM Na_2_HPO_4_ (pH 4.2) and 700 mM NaCl. The eluate was concentrated using a Pall Microsep Advance centrifugal filter unit (MWCO = 1000 Da) and purified by size-exclusion chromatography on a HiLoad 16/600 Superdex 30 pg column equilibrated with 50 mM Na_2_HPO_4_ (pH 4.2) and 0.15 M NaCl buffer. The toxin was further purified by reverse-phase (RP) HPLC on a Jupiter 300 C5 column (Phenomenex). Bs-Tx7-containing fractions were lyophilized and stored at −80 °C. The purity and molecular mass of the protein were verified by subsequent HPLC–UV/MS measurements (Fig. S3).

The protein concentration of the purified CTX and Bs-Tx7 samples was determined based on absorbance measurements at 214 nm using an online protein extinction coefficient calculator (https://bestsel.elte.hu/extcoeff.php) according to the method of Kuipers and Gruppen (35). CTX aliquots were stored and lyophilized at −80 °C.

### Fluorescently labeled test substances

CTX-Cy5 was synthesized at Vichem Chemie Research Ltd by conjugation reaction of CTX with cyanine 5 *N*-hydroxysuccinimide ester (Lumiprobe). The reacted sample was purified to homogeneity using RP chromatography (RP-HPLC–UV/MS) by collecting the fractions of a peak containing monoconjugated CTX-Cy5. According to structure elucidation studies by analytical RP-HPLC followed by tandem mass spectrometry, the CTX-Cy5 product used in the present studies proved to be a mixture of monoconjugate labeling the three lysine residues at positions 15, 23, and 27 in approximate amounts of 20%, 38%, and 42%, respectively.

CTX-A488 was also synthesized by a similar process, except that the reactive dye compound was Alexa Fluor 488 tetrafluorophenyl ester (ThermoFisher Scientific).

### Production of VEGF-A165

The DNA sequence encoding the mature form of human VEGF-A (VEGF-A165; amino acids: 27-191 of NP_001165100.1) was amplified from the complementary DNA of U87MG cells in a way that SplB recognition site was introduced upstream of the coding sequence. This segment was cloned into pET28a(+) vector (Novagen) using the restriction enzymes NdeI and XhoI (New England Biolabs) to yield a construct encoding VEGF-A165 with an N-terminal His tag that can be removed with SplB.

Shuffle T7 Express *E. coli* cells were transformed with the pET28-VEGF-A165 vector. Transformed cells were grown at 30 °C in LB medium supplemented with 25 μg/ml kanamycin until absorbance reached 0.8 at 600 nm. Protein expression was induced with 0.5 mM IPTG at 18 °C overnight. Harvested cells were resuspended in 50 mM Tris–HCl (pH 8.0), 300 mM NaCl, 1 mM PMSF, and disrupted by sonication. Cell debris was removed by centrifugation at 48,000*g*, and the supernatant was applied to BioRad Nuvia IMAC Resin equilibrated with 50 mM Tris–HCl (pH 8.0), 300 mM NaCl, and 20 mM imidazole. His6-tagged proteins were eluted with 250 mM imidazole. The eluate was dialyzed against 50 mM Tris–HCl (pH 8.9) and 30 mM NaCl buffer. The sample was subjected to anion exchange chromatography on a HiTrap Q HP column. The fraction corresponding to the dimer form of the His-tagged VEGF-A165 was dialyzed against 50 mM Tris–HCl (pH 8.5), 150 mM NaCl, concentrated using Amicon Ultra-15 centrifugal filter units, supplemented with 10% glycerol, and stored at −80 °C.

### Ig-coated bead test

The Ig-coated bead test is based on the principle of anchoring target proteins *via* target protein–specific rabbit IgG antibodies to MagnaBind Goat Anti-Rabbit IgG microbeads (ThermoFisher Scientific) factory-coated with goat anti-rabbit antibodies and binding a fluorophore-labeled ligand to the target protein followed by measurement of the fluorescence intensity of the beads by flow cytometry. Volumes of 1 μl microbeads (specified bead diameter = 1–4 μm) were pipetted in each reaction tube, and three rounds of washing were performed. Each round of washing comprised (re)suspending the beads in 2 ml Dulbecco’s PBS (DPBS; Sigma), placing the tubes in a magnetic concentrator (DynaMag-5; ThermoFisher Scientific), and discarding the supernatant after 10 min. Then the beads were incubated at 4 °C for 1 h in 50 μl DPBS containing a rabbit antibody against the tested target protein, diluted at 1:100. The used anti-MMP-2 antibody was a polyclonal antibody (Sigma; catalog no.: HPA001939), which was raised against a synthetic sequence at amino acid positions 445 to 575 of human MMP-2 protein (NP_004521.1). The recombinant MMP-2 proteins used were either the proenzyme expressed in human embryonic kidney 293 (Sigma, catalog no.: SAE 0174 or ProSpec, catalog no.: Enz-100, the latter His tagged at C terminus) or the mature active enzyme (amino acids: 110–660), expressed in *E. coli* (Sigma, catalog no.: SRP3118 or ProSpec, catalog no.: Enz-769, the latter His-tagged at N terminus). The anti-NRP1 antibody was obtained from ThermoFisher Scientific (catalog no.: PA5-96531). The NRP1 protein anchored to the beads was the recombinantly produced human NRP1 isoform b (NP_001019799.2) fused with a C-terminal His tag (Sino Biological; catalog no.: 10011-H08H). After the antibody coating, the beads were washed three times and incubated overnight at 4 °C in 50 μl DPBS containing the selected target protein at a concentration of 0.16 μM, which corresponded to 10 μg/ml for active MMP-2 (MW = 62 kDa). In the case of Ig-coated bead test, there was no need to apply any blocking incubation to eliminate nonspecific binding.

For testing fluorophore-labeled test substances (*e.g.*, CTX-Cy5 or CTX-A488), the target protein–coated beads were resuspended in 50 μl of test solution containing the specified concentration of the labeled test substance and incubated for 1 h at room temperature. The test solutions were prepared by diluting with DPBS a stock solution of 100 μM test substance dissolved in water. After incubation with the test solution, the beads were washed three times, resuspended in 2 ml DPBS, and were ready for flow cytometry measurement.

For competitive displacement experiments, the target protein–coated beads were resuspended in 50 μl test solution containing the specified concentration of the unlabeled test substance dissolved in DPBS and incubated for 1 h at room temperature. Then 50 μl DPBS solution containing 2 μM labeled CTX (CTX-Cy5 or CTX-A488) and the specified concentration of the unlabeled test substance were added to incubate the beads in 1 μM labeled CTX (displaced indicator ligand) and the specified concentration of the unlabeled test substance (displacing ligand). The beads were incubated for additional 1 h at room temperature, then washed three times, resuspended in 2 ml DPBS, and were ready for flow cytometry measurement.

Flow cytometric analysis was performed with a flow cytometer (MACSQuant Analyzer 10; Miltenyi Biotec) using the 635 nm red laser for excitation, and R1 channel for fluorescent intensity measurement of Cy5-labeled ligand binding, or the 488 nm blue laser for excitation, and B1 channel for fluorescent intensity measurement of A488-labeled ligand binding in pulse area mode. The aspiration volume was 200 μl per sample, with a setup for gentle mixing and 10,000 events collected per sample. The flow rate was set slow, with a maximum number of events of 500 per second. An inclusive elliptic gate was applied around the main bead population, which was set based on a forward scatter *versus* side scatter dot plot obtained with control beads so that events related to doublets and multiplets were excluded from the analysis. The gating was kept constant throughout all experiments with the same type of beads.

For quantitative characterization of binding of labeled ligands, the median fluorescence intensity of gated events was used. The results were expressed in terms of RFI values normalized to negative control beads. Negative controls were beads unexposed to target proteins but subjected to staining with 1 μM concentration of fluorophore-labeled CTX. The relative affinities of labeled test substances were assessed by plotting RFI values against log concentrations and determining half maximal effective (staining) concentrations (EC_50_) by sigmoidal curve fitting (Fig. 3).

In the displacement studies, the results were converted to percent inhibitions (percent displacements) of labeled ligand binding, and relative affinities of unlabeled test substances were assessed by plotting percent displacements against log concentrations and determining IC_50_ by sigmoidal curve fitting using “sigmoidal dose–response (variable slope) with least squares method” algorithm using the following equation:

$$Y=Bottom\;+\;\frac{Top-Bottom}{1+10^{\left(LogEC50-X\right)\;\cdot \;Hillslope}}$$

In GraphPad Prism 8 (GraphPad Software, Inc), the algorithm enabled determining 95% confidence intervals as well. Statistical significance of differences between EC_50_ or IC_50_ values from simultaneously plotted curve fittings was assessed by extra sum-of-squares F test applying alpha = 0.05.

### Cobalt-coated bead test

The cobalt-coated bead test was used for measurement of binding intensities of CTX-Cy5 or phage-displayed ligand proteins to various target proteins. The test is based on the principle of anchoring various His-tagged target proteins (receptors) to immobilized cobalt-coated His-Tag Isolation & Pulldown Dynabeads (ThermoFisher Scientific) and then binding a fluorophore-labeled ligand (or ligand-displaying phages subsequently stained) to the target protein, and measuring of the bead-bound fluorescence intensity by flow cytometry.

Volumes of 0.5 μl of beads (specified bead diameter = 1 μm) were pipetted in each reaction tube, and three rounds of washing were performed. Each round of washing comprised (re)suspending the beads in 2 ml Tris-buffered saline (TBS) solution (ThermoFisher Scientific) containing 0.05% (v/v) Tween-20 (washing solution), placing the tubes in a magnetic concentrator (DynaMag-5), and discarding the supernatant after 10 min. Then the beads were incubated overnight at 4 °C in 50 μl target protein solution. We applied all target proteins at identical molar concentration: 0.16 μM, which is equivalent with 10 μg/ml in the case of active MMP-2 (MW = 62 kDa). The target protein solutions were dissolved or diluted in TBS. Negative control beads were incubated simultaneously with TBS containing no target protein. The used target proteins were the following (with National Center for Biotechnology Information accession number [segment amino acid sequence]; His-tag position: C- or N-terminal; supplier; and catalog number listed in parentheses): MMP2 (NP_004521.1 [amino acids: 110–660]; N-; ProSpec, catalog no.: ENZ 769), NRP1 (NP_001019799.2 [amino acids: 2–644]; C-; Sino Biological; catalog no.: 10011-H08H); MMP-9 (NP_004985.2 [amino acids: 20–701]; C-; ProSpec; catalog no.: ENZ 1091), TIMP-2 (NP_003246.1 [amino acids: 27–220]; N-; ProSpec; catalog no.: ENZ-646), MMP 14 (NP_004986 [amino acids: 24–524]; C-; ThermoFisher; catalog no.: RP77533), ANX2 (NP_001002857.1 [amino acids: 1–339]; N-; ProSpec; catalog no.: PRO-777), α_v_β_3_ integrin (AAA52589.1 & NP_002196.4 heterodimer; both with C-terminal His tag; Native Antigen Company; catalog no.: REC31719-100), CLC-3 (NP_001820.2 [amino acids: 1–818]; C-; Creative Biomart; custom made by recombinant expression in *E. coli*), and human serum albumin (NP_000468.1 [amino acids: 25–609]; C-; Abcam; catalog no.: ab217817). Following target protein incubation, the beads were washed three times and then blocked by incubation with 100 μl Casein Blocking Solution (Sigma; catalog no.: B6429) for 1 h at room temperature, followed by three rounds of washing. The subsequent process was different for labeled ligand protein test substances and phage-displayed ligand proteins.

For testing fluorophore-labeled CTX (CTX-Cy5), the target protein–coated and casein-blocked beads were resuspended in 50 μl test solution containing the specified concentration of the labeled test substance and incubated for 1 h at room temperature. After incubation with the test solution, the beads were washed three times, resuspended in 2 ml washing solution, and were ready for flow cytometry measurement.

In the case of testing phage-displayed ligand proteins, the prepared beads were resuspended in the tested phage solution and incubated for 1 h at room temperature. The phages were dissolved in a phage and antibody incubation buffer, TBS containing 0.5% bovine serum albumin, and 0.05% (v/v) Tween-20 (hereafter abbreviated as TBT). After phage exposure, the beads were washed three times and then incubated for 30 min at 4 °C in 50 μl TBT containing mouse monoclonal anti-M13 antibody (Sino Biological; catalog no.: 11973-MM05) at a dilution of 1:200. Then the beads were washed three times and incubated for 30 min at 4 °C in 50 μl TBT containing Cy5-labeled goat antimouse (Abcam; catalog no.: ab6563) secondary antibody diluted at 1:1000. After three rounds of final washing, the beads were resuspended in 2 ml washing solution and were ready for flow cytometry measurement.

Flow cytometric analysis was carried out in the same manner as described previously at the Ig-coated bead test, quantitatively characterizing the intensity of binding by the RFI values calculated from median fluorescence intensities of beads.

In the cobalt-coated bead phage binding validation experiment, the matching target protein for DX-88-displaying phages was recombinant human plasma kallikrein/KLKB1 (R&D Systems), Gly20-Ala638, with a C-terminal His tag.

### Preparation of phages for phage-displayed ligand peptide binding measurements

Phage display test solutions were prepared according to routine phage display protocols (36). Phosphorylated and annealed oligonucleotides encoding the peptide construct were ligated into linearized pAS62 phagemid vector (37) at a vector/insert ratio of 1:3. The final phagemid construct harbored a signal sequence followed by the peptide of interest, a linker sequence (GSASSATR), and the C-terminal part of the P3 coat protein (amino acids: 216-424 of NP_510891.1). Chemically competent XL1-Blue *E. coli* cells (catalog no.: 2022249; Agilent) were transformed with the ligation product and then streaked onto ampicillin (100 μg/ml) containing LB/Amp plates and incubated for 16 h at 37 °C. A few clones were sequenced, and a single colony with confirmed target sequence was inoculated into 3 ml 2YT medium supplemented with 100 μg/ml ampicillin and incubated at 37 °C until the culture reached midlog phase. The culture was infected with M13KO7 helper phage (New England Biolabs) (phage/cell ratio 10:1) and was incubated for 30 min at 37 °C. The 3 ml culture was transferred into 200 ml 2YT medium, supplemented with 100 μg/ml ampicillin and 25 μg/ml kanamycin, and incubated overnight at 37 °C. The overnight cell culture (200 ml) was pelleted by centrifugation at 8000*g* for 10 min in 400 ml centrifuge tubes at 4 °C. The supernatant was transferred into a fresh 400 ml centrifuge tube containing 40 ml PEG/NaCl (200 g/l PEG-8000 and 146.1 g/l NaCl) and then was mixed thoroughly and incubated at room temperature for 20 min. Phages were pelleted by centrifugation at 18,000*g* for 15 min at room temperature. After discarding the supernatant, the tubes were centrifuged again at 1000 rpm for 1 min, then phages were solubilized in 4 ml phage resuspension buffer (TBT), and all insoluble materials were removed by centrifugation at 18,000*g* for 10 min at 4 °C. The supernatant was transferred into four 1.5 ml centrifuge tubes (4 × 1 ml) and precipitated again with 200 to 200 μl PEG/NaCl and was incubated for an additional 5 min before centrifuged again at 12,000*g*. The pellets were resuspended in low volumes (150–300 μl) of phage resuspension buffer and combined into a single phage solution. The particle concentrations of phage solutions were determined using a NanoDrop One C (Thermo Scientific) spectrophotometer. The phage particle concentration was assessed according to the formula: (OD_268_-OD_320_) × 5 × 10^12^ particles/ml (36); and set to target concentration by diluting with TBT.

### MMP-2 inhibition assay with DQ-gelatin substrate

The MMP-2 enzyme inhibitory effect of CTX and positive control ilomastat was assessed by the fluorogenic DQ-gelatin assay in a similar way as described for assessing MMP-9 inhibitors (38). The assay was performed using fluorogenic dye-quenched (DQ)-gelatin from pig skin (ThermoFisher Scientific) as substrate and recombinant human MMP-2 enzyme (Sigma; catalog no.: SRP3118), dissolved in water (100 μg/ml), and diluted further in reaction buffer (50 mM Tris, 150 mM NaCl, 5 mM CaCl_2,_ pH = 7.4). The assay (and control) reactions were composed by loading 50 μl enzyme (or buffer), 40 μl inhibitor (or buffer), and 10 μl substrate (or buffer) into wells of a 96-well plate (PS, black, half-area, clear-bottom; Greiner Bio-One) at concentrations needed for the stated final concentrations. The enzyme plus inhibitor mix was incubated at room temperature for 30 min before addition of the substrate. Immediately thereafter, the plate was placed in a fluorescence plate reader (SpectraMax iD3; Molecular Devices), and fluorescence was measured every 15 min for 3 h (*K*_*M*_ measurements) or every 5 min for 1 h (inhibitor measurements) at 37 °C (λ_excitation_ = 484 nm and λ_emission_ = 525 nm). *K*_*M*_ was determined by measuring V_0_ at a series of substrate concentrations (1.25, 2.5, 5, 10, 20, and 40 μg/ml) and enzyme concentrations of 2.5, 5, and 10 nM. Thereafter, the lowest enzyme concentration with reasonably good signal-to-noise ratio (2.5 nM) and a substrate concentration (3.5 μg/ml) approximately equivalent with the determined *K*_*M*_ value (3.4 μg/ml, *i.e.*, 34 nmol/l as calculated with approximate MW of 100,000 g/mol for DQ gelatin) were applied in the enzyme inhibition study. For the inhibitor activity measurements, eight-point concentration series were prepared with 3.16-fold (square root 10-fold) dilution steps and tested in at least quadruplicates along with positive (no inhibitor) and negative (no enzyme) controls. Initial reaction velocities (V_0_) were determined from change of fluorescence between 0 min and 30 min repeatedly measured in 5-min-long cycles and evaluated as slopes of regression lines fitted on intensity measurements. These values were converted to percentages taking the average of positive control slopes as 100% and negative control slopes as 0%. Statistical significance of difference from positive control was tested by ANOVA followed by Dunnett test.

### MMP-2 inhibition assay with an oligopeptide substrate

The assay was performed using a fluorescence-quenched MMP-specific peptide substrate FS-6 (Sigma; catalog no.: SCP0193, *K*_*M*_ >30 μM) (20). Recombinantly produced human proMMP2 enzyme, dissolved in reaction buffer (70 mM Tris [pH 8.0], 150 mM NaCl, 5 mM CaCl_2_, 1 μM ZnCl_2_, and 0.05% Brij-35) at a concentration of 500 nM, was activated by addition of 1 mM APMA and incubated at 37 °C for 1 h. The assay reaction was then composed by adding 0.5 μl activated enzyme to yield a final concentration of 2.5 nM, 94.5 μl test substance (inhibitor) in reaction buffer, and finally 5 μl substrate to reach a final concentration of 50 μM in wells of a 96-well plate. The enzyme plus inhibitor mix was incubated at 37 °C for 30 min before the addition of the substrate and start of the fluorescence intensity measurements. The change in the fluorescence intensity was recorded in kinetic mode (λ_excitation_ = 380 nm, λ_emission_ = 460 nm) using a fluorescence plate reader (SpectraMax iD3; Molecular Devices). The effect of rCTX on the enzymatic activity of MMP2 was investigated at a test concentration of 30 μM, along with positive (no test substance) and negative (no enzyme) controls. Furthermore, ilomastat was tested as an MMP inhibitor positive control at two selected concentrations to confirm sensitivity of the assay for MMP-2 inhibition. Initial reaction velocities (V_0_) were determined from the apparently linear change of fluorescence between 0 min and 30 min in the reader and evaluated as slopes of regression lines fitted on intensity measurements repeated in 33-s-long cycles. These values were converted to percentages taking the average of positive control slopes as 100% and negative control slopes as 0%. Statistical significance of difference from positive control was tested by ANOVA followed by Dunnett test.

## Data availability

All main text data are in this article. Supporting information are in the corresponding Supporting Information document. Correspondence and requests for materials should be addressed to the corresponding author (sandor.farkas@vrgtherapeutics.com).

## Supporting information

This article contains supporting information.

## Conflict of interest

The authors declare that they have no conflicts of interest with the contents of this article.
