# Supplementary material for: Chlorotoxin binds to both matrix metalloproteinase 2 and neuropilin 1
Source: J Biol Chem. 2023 Jun 30;299(9):104998. doi: 10.1016/j.jbc.2023.104998 (PMC10477481; doi:10.1016/j.jbc.2023.104998)
Supplement: Supporting Figures S1–S3 [file mmc1.pdf]

SUPPORTING INFORMATION

Chlorotoxin binds to both matrix metalloproteinase 2 and neuropilin 1

Sándor Farkas, Daniel Cioca, József Murányi, Péter Hornyák, Attila Brunyánszki, Patrik Szekér, Eszter Boros, Patrik Horváth, Zoltán Hujber, Gábor Z. Rácz, Noémi Nagy, Rebeka Tóth, László Nyitray, Zalán Péterfi

LC-MS analysis of rCTX, mCTX and Bs-Tx7

LC-MS analysis was performed on a Waters Select Series Cyclic ion mobility mass spectrometer or a Waters Acquity RDa detector coupled to a Waters Acquity I-Class UPLC system.Chromatographic separations were conducted on a Waters Acquity UPLC BEH300 C4 column (2.1x150 mm, 1.7 µm) set at 80°C using a water/acetonitrile solvent system supplemented with 0.1% trifluoroacetic acid run at 0.2 ml/min. Lyophilized peptides were dissolved in 5% acetonitrile, 10 µl of each sample was injected and a linear gradient was applied for eluting the peptides.

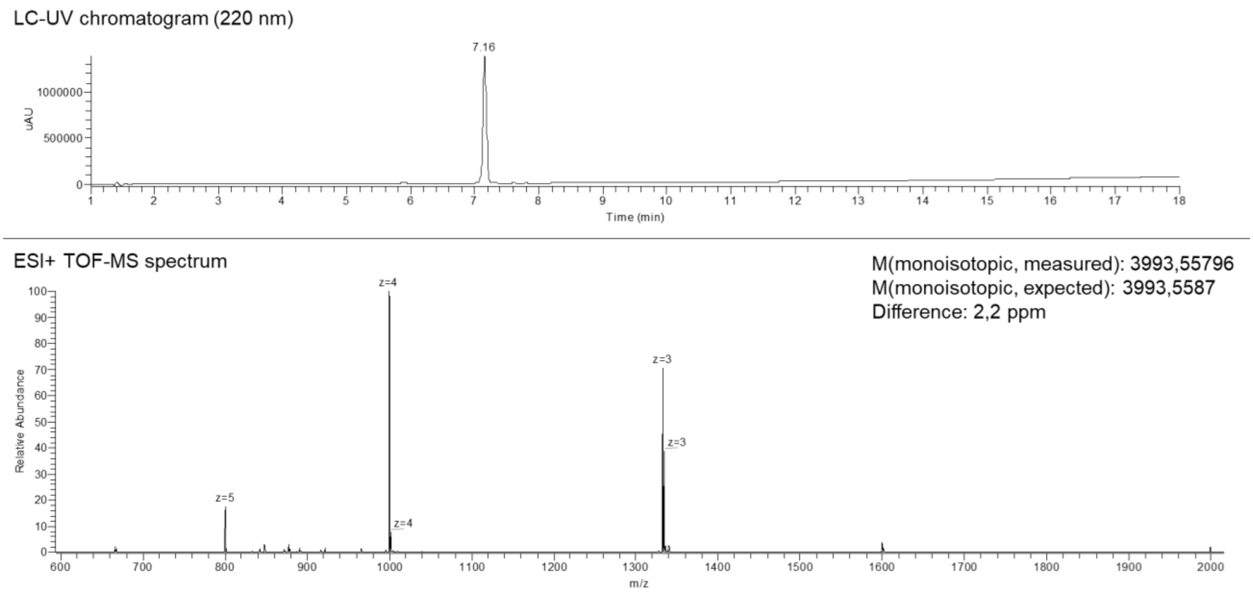

Fig. S1. HPLC-UV chromatogram and MS spectrum of purified recombinant chlorotoxin (rCTX)

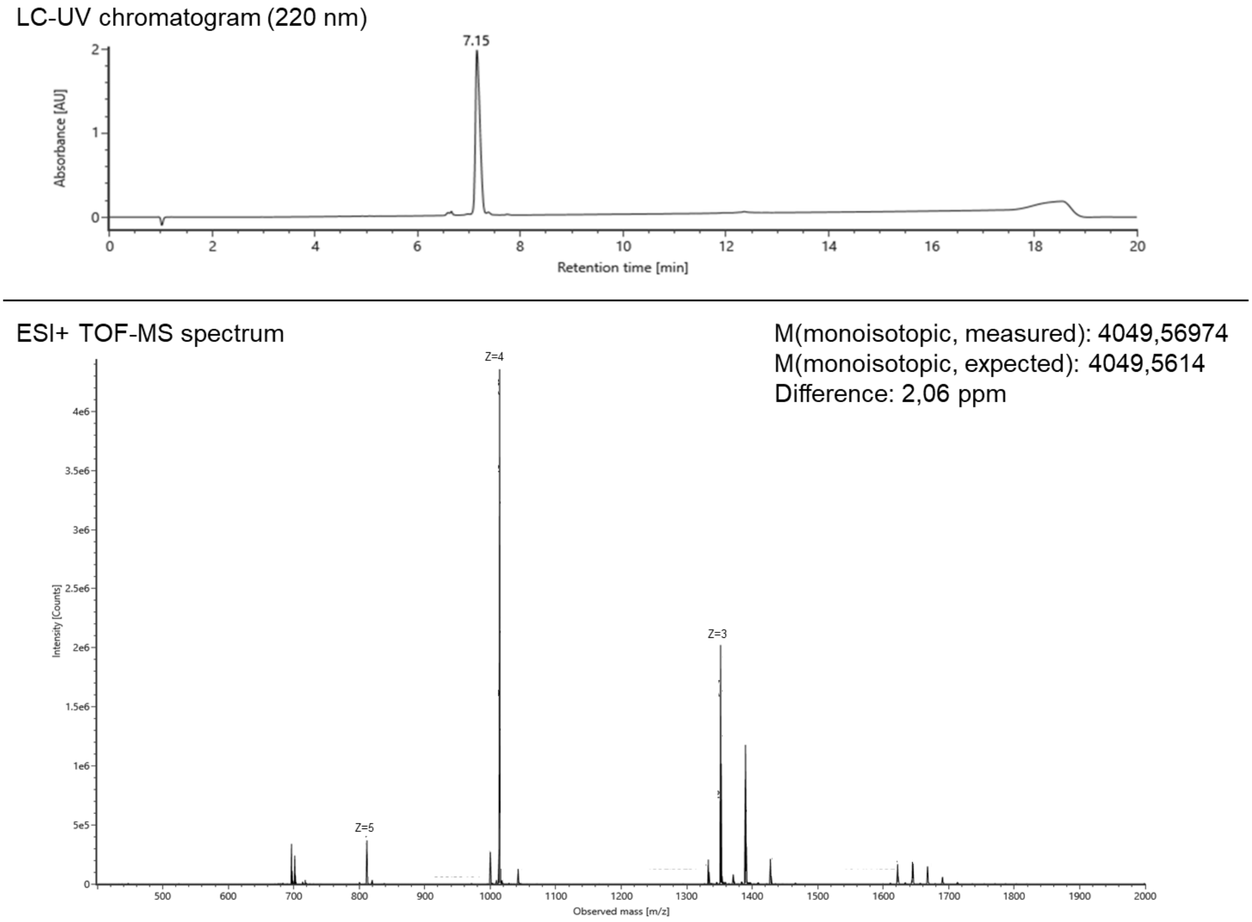

Fig. S2. HPLC-UV chromatogram and MS spectrum of purified recombinant monolysine (K15R, K23R) mutant chlorotoxin (mCTX).

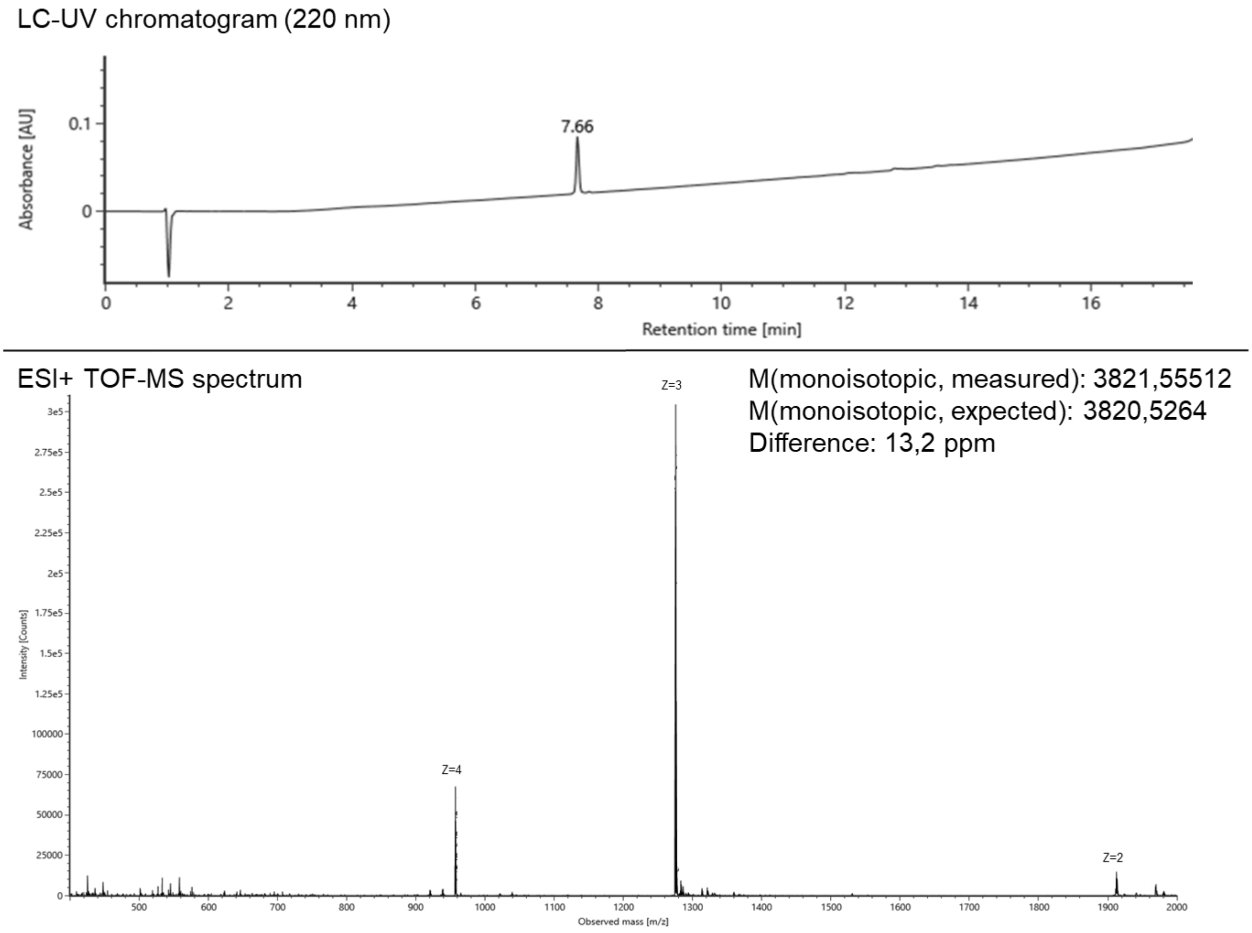

Fig. S3. HPLC-UV chromatogram and MS spectrum of purified recombinant Bs-Tx7
